# Notes and Notices

**Published:** 1869-01

**Authors:** 


### NOTES AND NOTICES.

American Houses. A Variety of Original Designs for Rural Buildings. Illustrated by Twenty-six colored Engravings, with Descriptive References. By Samuel Sloan, Architect. Philadelphia: Henry Carey Baird, 406 Walnut street. Sent free of postage on receipt of $2.50.

The object of this book, as announced by the author, is to present a number of designs in an attractive dress, that may either serve as models to build from, or criterions by which the projector may judge of the relative quality and merits of his intended edifice. It will prove of value to such as need some guide in forming a judgment upon designs, and who, meditating the erection of rural buildings, wish for some hints upon the subject before consulting an architect. Architects also will find useful studies in this book, and we especially recommend it to beginners and students.

The Publishing House of Lee & Shepard, Boston, Mass., has just issued the following seasonable works:

Philosophy of Domestic Life, by W. H. Byford, M.D., of Chicago, Ill. 174 pp. 12mo. Legal Relations; Family Relations; Marriage; Errors of Motive in Marrying; Ethics of Married Life; Family Government; Education, &c.

The Mimic Stage, being a Series of Dramas, Comedies, Burlesques, and Farces for public exhibitions and private theatricals, by George M. Baker. 290 pp. 12mo.

